# (3,6-Dimeth­oxy-2-naphth­yl)(4-fluoro­benzo­yl)methanone

**DOI:** 10.1107/S1600536810006859

**Published:** 2010-02-27

**Authors:** Shoji Watanabe, Toyokazu Muto, Atsushi Nagasawa, Akiko Okamoto, Noriyuki Yonezawa

**Affiliations:** a Department of Organic and Polymer Materials Chemistry, Tokyo University of Agriculture & Technology, Koganei, Tokyo 184-8588, Japan

## Abstract

In the title compound, C_19_H_15_FO_3_, the dihedral angle between the naphthalene ring system and the benzene ring is 62.93 (5)°. The bridging carbonyl C—C(=O)—C plane makes dihedral angles of 45.55 (6) and 28.62 (7)°, respectively, with the naphthalene ring system and the benzene ring. Weak inter­molecular C—H⋯O hydrogen bonds and C—H⋯π inter­actions stabilize the crystal packing.

## Related literature

For general background to the regioselective formation of *peri*-aroylnaphthalene compounds, see: Okamoto & Yonezawa (2009 ▶). For related structures, see: Hijikata *et al.* (2010 ▶); Mitsui *et al.* (2008 ▶); Nakaema *et al.* (2007 ▶, 2008 ▶); Watanabe *et al.* (2010*a* ▶,*b* ▶).
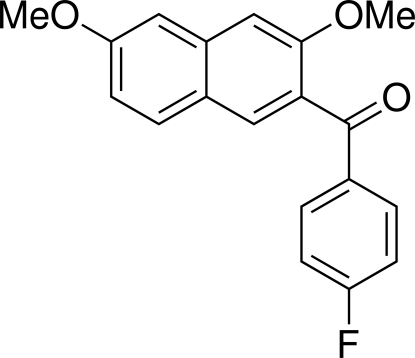


## Experimental

### 

#### Crystal data

C_19_H_15_FO_3_
*M* *_r_* = 310.31
Monoclinic, 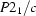

*a* = 8.3690 (2) Å
*b* = 19.7603 (5) Å
*c* = 9.3897 (2) Å
β = 105.126 (2)°
*V* = 1499.01 (6) Å^3^
*Z* = 4
Cu *K*α radiation
μ = 0.84 mm^−1^
*T* = 193 K
0.55 × 0.50 × 0.45 mm

#### Data collection

Rigaku R-AXIS RAPID diffractometer
Absorption correction: numerical (*NUMABS*; Higashi, 1999 ▶) *T*_min_ = 0.657, *T*_max_ = 0.705
26258 measured reflections
2738 independent reflections
2530 reflections with *I* > 2σ(*I*)
*R*_int_ = 0.023

#### Refinement

*R*[*F* ^2^ > 2σ(*F* ^2^)] = 0.032
*wR*(*F* ^2^) = 0.090
*S* = 1.02
2738 reflections
211 parameters
H-atom parameters constrained
Δρ_max_ = 0.20 e Å^−3^
Δρ_min_ = −0.11 e Å^−3^

### 

Data collection: *PROCESS-AUTO* (Rigaku, 1998 ▶); cell refinement: *PROCESS-AUTO*; data reduction: *CrystalStructure* (Rigaku/MSC, 2004 ▶); program(s) used to solve structure: *SIR2004* (Burla *et al.*, 2005 ▶); program(s) used to refine structure: *SHELXL97* (Sheldrick, 2008 ▶); molecular graphics: *ORTEPIII* (Burnett & Johnson, 1996 ▶); software used to prepare material for publication: *SHELXL97*.

## Supplementary Material

Crystal structure: contains datablocks I, global. DOI: 10.1107/S1600536810006859/bt5198sup1.cif

Structure factors: contains datablocks I. DOI: 10.1107/S1600536810006859/bt5198Isup2.hkl

Additional supplementary materials:  crystallographic information; 3D view; checkCIF report

## Figures and Tables

**Table 1:** Hydrogen-bond geometry (Å, °) *Cg*1 is the centroid of the C1–C5/C10 ring.

*D*—H⋯*A*	*D*—H	H⋯*A*	*D*⋯*A*	*D*—H⋯*A*
C18—H18*B*⋯*Cg*1^i^	0.98	2.85	3.7479 (14)	152
C17—H17⋯O1^ii^	0.95	2.55	3.2930 (16)	136
C18—H18*A*⋯O3^iii^	0.98	2.39	3.3603 (16)	169

Symmetry codes: (i) 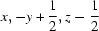; (ii) 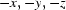; (iii) 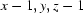.

**Fig. 1.**
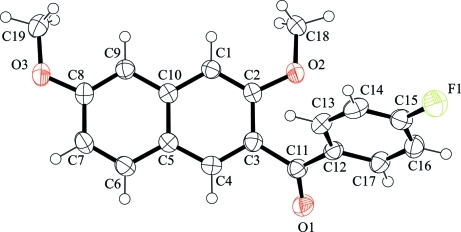
Molecular structure of (I), with the atom-labeling scheme and displacement ellipsoids drawn at the 50 % probability level.

**Fig. 2.**
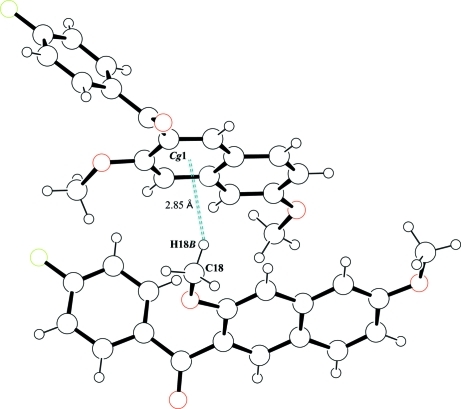
C—H···π interactions (green dotted lines).

**Fig. 3.**
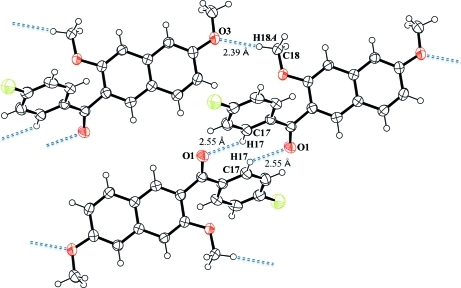
Two types of intermolecular weak C—H···O interactions (blue dotted lines).

## supplementary crystallographic information

### Comment

In the course of our study on electrophilic aromatic aroylation of 2,7-dimethoxynaphthalene, *peri*-aroylnaphthalene compounds have proven to be formed regioselectively with the aid of suitable acidic mediators (Okamoto & Yonezawa, 2009). The aroyl groups at the 1,8-positions of the naphthalene rings in these compounds are twisted almost perpendicularly but the benzene ring moieties of the aroyl groups tilt slightly toward the exo sides of the naphthalene rings.

Recently, we reported the structures of 1,8-diaroyl-2,7-dimethoxynaphthalenes, i. e., 1,8-bis(4-chlorobenzoyl)-2,7-dimethoxynaphthalene (Nakaema *et al.*, 2007), bis(4-bromobenzoyl)(2,7-dimethoxynaphthalene-1,8-diyl)dimethanone (Watanabe *et al.*, 2010*a*). In addition, the crystal structural analysis of 1-aroyl-2,7-dimethoxynaphthalenes, i. e., methyl 4-(2,7-dimethoxy-1-naphthoyl)benzoate (Hijikata *et al.*, 2010) and 1-(4-nitorobenzoyl)-2,7-dimethoxynaphthalene (Watanabe *et al.* 2010*b*), also has revealed essentially the same non-coplanar structure as the 1,8-diaroylated naphthalenes.

Furthermore, the structure of 3-aroyl-2,7-dimetoxynaphthalenes such as 2-(4-chlorobenzoyl)-3,6-dimethoxynaphthalene (Nakaema *et al.*, 2008), which are generally regarded to be thermodynamically more stable than the corresponding 1-positioned isomeric molecules, 1-(4-chlorobenzoyl)-2,7-dimethoxynaphthalene (Mitsui *et al.*, 2008), has been also studied. As a part of our ongoing work on the formation reaction and the structure of the aroylated naphthalene derivatives, the synthesis and crystal structure of (I), a 3-monoaroylnaphthalene bearing fluoro group, is discussed in this report. (I) was prepared by electrophilic aromatic aroylation reaction of 2,7-dimethoxynaphthalene with 4-fluorobenzoic acid.

An *ORTEPIII* (Burnett & Johnson, 1996) plot of (I) is displayed in Fig. 1. The 4-fluorophenyl group is twisted away from the attached naphthalene ring. The dihedral angle between the best planes of the fluorophenyl ring (C12—C17) and the naphthalene ring (C1—C10) is 62.93 (5)°. The bridging carbonyl plane (O1—C11—C3—C12) makes relatively large dihedral angle of 45.55 (6)° with the naphthalene ring (C1—C10) [C4—C3—C11—O1 torsion angle = 43.90 (17)°], whereas it makes rather small one of 28.62 (7)° with 4-fluorophenyl ring (C12—C17) [O1—C11—C12—C17 torsion angle = 28.69 (18)°].

Molecules are linked by C—H···π interactions (Fig. 2). The methyl group acts as an hydrogen-bond donor and π system of the naphthalene ring [C1—C5/C10 ring (with centroid *Cg*1)] of an adjacent molecule acts as an accepter (C18—H18*B*···π).

The crystal packing is additionally stabilized by two types of intermolecular weak C—H···O hydrogen bondings: One is between the aromatic hydrogen (H17) at meta position to the F group, and the carbonyl oxygen (O1) (Fig.3, Table 1). The other is between an hydrogen (H18*A*) of the 2-methoxy group which is situated adjacent to the fluorophenyl group, and the ethereal oxygen (O3) of the 7-methoxy group in a neighboring molecule .

### Experimental

The title compound was prepared by treatment of a mixture of 2,7-dimethoxynaphthalene (0.20 mmol) and 4-fluoroobenzoic acid (0.21 mmol) with phosphorus pentoxide–methanesulfonic acid mixture (P_2_O_5_–MsOH [1/10 w/w]; 0.44 ml) at 60°C for 24 hours followed by a typical work-up procedure (30% yield; Okamoto & Yonezawa, 2009). Colorless block single crystals suitable for X-ray diffraction were obtained by recrystallization from chloroform.

### Refinement

All the H atoms were found in difference maps and were subsequently refined as riding atoms, with C—H = 0.95 (aromatic) and 0.98 (methyl) Å, and U_iso_(H) = 1.2U_eq_(C).

### Crystal data

**Table:**

C_19_H_15_FO_3_	*F*(000) = 648
*M**_r_* = 310.31	*D*_x_ = 1.375 Mg m^−^^3^
Monoclinic, *P*2_1_/*c*	Melting point = 409.7–410.3 K
Hall symbol: -P 2ybc	Cu *K*α radiation, λ = 1.54187 Å
*a* = 8.3690 (2) Å	Cell parameters from 20173 reflections
*b* = 19.7603 (5) Å	θ = 4.5–68.2°
*c* = 9.3897 (2) Å	µ = 0.84 mm^−^^1^
β = 105.126 (2)°	*T* = 193 K
*V* = 1499.01 (6) Å^3^	Block, colorless
*Z* = 4	0.55 × 0.50 × 0.45 mm

### Data collection

**Table:**

Rigaku R-AXIS RAPID diffractometer	2738 independent reflections
Radiation source: fine-focus sealed tube	2530 reflections with *I* > 2σ(*I*)
graphite	*R*_int_ = 0.023
Detector resolution: 10.00 pixels mm^-1^	θ_max_ = 68.2°, θ_min_ = 4.5°
ω scans	*h* = −10→9
Absorption correction: numerical (*NUMABS*; Higashi, 1999)	*k* = −23→23
*T*_min_ = 0.657, *T*_max_ = 0.705	*l* = −11→11
26258 measured reflections	

### Refinement

**Table:**

Refinement on *F*^2^	Secondary atom site location: difference Fourier map
Least-squares matrix: full	Hydrogen site location: inferred from neighbouring sites
*R*[*F*^2^ > 2σ(*F*^2^)] = 0.032	H-atom parameters constrained
*wR*(*F*^2^) = 0.090	*w* = 1/[σ^2^(*F*_o_^2^) + (0.0489*P*)^2^ + 0.3826*P*] where *P* = (*F*_o_^2^ + 2*F*_c_^2^)/3
*S* = 1.02	(Δ/σ)_max_ < 0.001
2738 reflections	Δρ_max_ = 0.20 e Å^−^^3^
211 parameters	Δρ_min_ = −0.11 e Å^−^^3^
0 restraints	Extinction correction: *SHELXL97* (Sheldrick, 2008), Fc^*^=kFc[1+0.001xFc^2^λ^3^/sin(2θ)]^-1/4^
Primary atom site location: structure-invariant direct methods	Extinction coefficient: 0.0068 (5)

### Special details

**Table:**

Geometry. All esds (except the esd in the dihedral angle between two l.s. planes) are estimated using the full covariance matrix. The cell esds are taken into account individually in the estimation of esds in distances, angles and torsion angles; correlations between esds in cell parameters are only used when they are defined by crystal symmetry. An approximate (isotropic) treatment of cell esds is used for estimating esds involving l.s. planes.
Refinement. Refinement of *F*^2^ against ALL reflections. The weighted *R*-factor wR and goodness of fit *S* are based on *F*^2^, conventional *R*-factors *R* are based on *F*, with *F* set to zero for negative *F*^2^. The threshold expression of *F*^2^ > σ(*F*^2^) is used only for calculating *R*-factors(gt) etc. and is not relevant to the choice of reflections for refinement. *R*-factors based on *F*^2^ are statistically about twice as large as those based on *F*, and *R*- factors based on ALL data will be even larger.

### Fractional atomic coordinates and isotropic or equivalent isotropic displacement parameters (Å^2^)

**Table:**

	*x*	*y*	*z*	*U*_iso_*/*U*_eq_	
F1	0.23655 (12)	0.05261 (4)	−0.47975 (8)	0.0590 (3)	
O1	0.24071 (11)	0.01533 (5)	0.18556 (10)	0.0455 (2)	
O2	0.27326 (11)	0.19750 (4)	0.05828 (9)	0.0398 (2)	
O3	0.94318 (11)	0.28084 (5)	0.70653 (10)	0.0440 (2)	
C1	0.49935 (15)	0.22971 (6)	0.26543 (13)	0.0323 (3)	
H1	0.4904	0.2759	0.2362	0.039*	
C2	0.39672 (14)	0.18266 (6)	0.18062 (12)	0.0320 (3)	
C3	0.41037 (14)	0.11285 (6)	0.22130 (12)	0.0324 (3)	
C4	0.52148 (14)	0.09433 (6)	0.35119 (13)	0.0336 (3)	
H4	0.5262	0.0483	0.3813	0.040*	
C5	0.62836 (14)	0.14138 (6)	0.44084 (12)	0.0318 (3)	
C6	0.74594 (15)	0.12300 (6)	0.57403 (13)	0.0365 (3)	
H6	0.7542	0.0771	0.6052	0.044*	
C7	0.84681 (15)	0.17022 (6)	0.65743 (13)	0.0384 (3)	
H7	0.9247	0.1571	0.7461	0.046*	
C8	0.83613 (14)	0.23878 (6)	0.61265 (13)	0.0349 (3)	
C9	0.72528 (14)	0.25877 (6)	0.48456 (13)	0.0329 (3)	
H9	0.7199	0.3049	0.4551	0.039*	
C10	0.61869 (14)	0.21041 (6)	0.39610 (12)	0.0306 (3)	
C11	0.30651 (14)	0.05923 (6)	0.12869 (13)	0.0342 (3)	
C12	0.29002 (14)	0.05826 (6)	−0.03341 (13)	0.0325 (3)	
C13	0.41406 (15)	0.08347 (6)	−0.09276 (14)	0.0377 (3)	
H13	0.5108	0.1027	−0.0294	0.045*	
C14	0.39778 (17)	0.08070 (6)	−0.24310 (15)	0.0428 (3)	
H14	0.4831	0.0969	−0.2839	0.051*	
C15	0.25461 (17)	0.05384 (6)	−0.33183 (13)	0.0409 (3)	
C16	0.13017 (16)	0.02778 (7)	−0.27807 (14)	0.0420 (3)	
H16	0.0334	0.0090	−0.3424	0.050*	
C17	0.14990 (15)	0.02967 (6)	−0.12746 (14)	0.0382 (3)	
H17	0.0663	0.0111	−0.0874	0.046*	
C18	0.23490 (16)	0.26755 (6)	0.02792 (14)	0.0402 (3)	
H18A	0.1387	0.2715	−0.0575	0.048*	
H18B	0.3300	0.2904	0.0065	0.048*	
H18C	0.2098	0.2887	0.1140	0.048*	
C19	0.92919 (19)	0.35155 (7)	0.67676 (16)	0.0505 (4)	
H19A	1.0101	0.3760	0.7539	0.061*	
H19B	0.8173	0.3668	0.6752	0.061*	
H19C	0.9506	0.3606	0.5808	0.061*	

### Atomic displacement parameters (Å^2^)

**Table:**

	*U*^11^	*U*^22^	*U*^33^	*U*^12^	*U*^13^	*U*^23^
F1	0.0822 (6)	0.0620 (5)	0.0351 (4)	−0.0059 (4)	0.0196 (4)	−0.0056 (4)
O1	0.0478 (5)	0.0476 (5)	0.0395 (5)	−0.0152 (4)	0.0088 (4)	0.0038 (4)
O2	0.0411 (5)	0.0351 (5)	0.0353 (4)	0.0021 (4)	−0.0042 (4)	−0.0002 (3)
O3	0.0421 (5)	0.0433 (5)	0.0387 (5)	−0.0027 (4)	−0.0039 (4)	−0.0046 (4)
C1	0.0351 (6)	0.0297 (6)	0.0313 (6)	0.0017 (5)	0.0071 (5)	0.0016 (4)
C2	0.0311 (6)	0.0352 (6)	0.0284 (5)	0.0017 (5)	0.0056 (4)	0.0004 (5)
C3	0.0319 (6)	0.0340 (6)	0.0315 (6)	−0.0009 (5)	0.0085 (5)	−0.0006 (5)
C4	0.0359 (6)	0.0310 (6)	0.0346 (6)	0.0009 (5)	0.0104 (5)	0.0021 (5)
C5	0.0314 (6)	0.0334 (6)	0.0310 (6)	0.0023 (4)	0.0090 (5)	0.0009 (4)
C6	0.0382 (6)	0.0353 (6)	0.0348 (6)	0.0044 (5)	0.0073 (5)	0.0043 (5)
C7	0.0368 (6)	0.0433 (7)	0.0311 (6)	0.0059 (5)	0.0015 (5)	0.0031 (5)
C8	0.0314 (6)	0.0400 (7)	0.0319 (6)	0.0005 (5)	0.0058 (5)	−0.0045 (5)
C9	0.0335 (6)	0.0321 (6)	0.0325 (6)	0.0012 (5)	0.0078 (5)	−0.0004 (5)
C10	0.0298 (6)	0.0333 (6)	0.0295 (6)	0.0017 (4)	0.0092 (4)	−0.0001 (4)
C11	0.0316 (6)	0.0320 (6)	0.0383 (6)	−0.0011 (5)	0.0081 (5)	0.0018 (5)
C12	0.0336 (6)	0.0273 (5)	0.0364 (6)	−0.0008 (4)	0.0085 (5)	−0.0014 (4)
C13	0.0374 (6)	0.0334 (6)	0.0427 (7)	−0.0064 (5)	0.0111 (5)	−0.0051 (5)
C14	0.0520 (8)	0.0349 (6)	0.0479 (7)	−0.0058 (5)	0.0247 (6)	−0.0038 (5)
C15	0.0562 (8)	0.0339 (6)	0.0336 (6)	0.0034 (5)	0.0136 (6)	−0.0033 (5)
C16	0.0415 (7)	0.0423 (7)	0.0389 (7)	−0.0020 (5)	0.0048 (5)	−0.0063 (5)
C17	0.0356 (6)	0.0387 (6)	0.0403 (6)	−0.0061 (5)	0.0103 (5)	−0.0026 (5)
C18	0.0400 (7)	0.0370 (6)	0.0376 (7)	0.0032 (5)	−0.0007 (5)	0.0047 (5)
C19	0.0513 (8)	0.0409 (7)	0.0509 (8)	−0.0057 (6)	−0.0018 (6)	−0.0071 (6)

### Geometric parameters (Å, °)

**Table:**

F1—C15	1.3575 (14)	C8—C9	1.3715 (16)
O1—C11	1.2224 (15)	C9—C10	1.4186 (16)
O2—C2	1.3615 (14)	C9—H9	0.9500
O2—C18	1.4327 (15)	C11—C12	1.4922 (16)
O3—C8	1.3632 (14)	C12—C17	1.3906 (17)
O3—C19	1.4238 (16)	C12—C13	1.3922 (17)
C1—C2	1.3709 (16)	C13—C14	1.3835 (18)
C1—C10	1.4174 (16)	C13—H13	0.9500
C1—H1	0.9500	C14—C15	1.3750 (19)
C2—C3	1.4279 (16)	C14—H14	0.9500
C3—C4	1.3763 (16)	C15—C16	1.3708 (19)
C3—C11	1.4965 (16)	C16—C17	1.3808 (18)
C4—C5	1.4072 (16)	C16—H16	0.9500
C4—H4	0.9500	C17—H17	0.9500
C5—C6	1.4219 (16)	C18—H18A	0.9800
C5—C10	1.4232 (16)	C18—H18B	0.9800
C6—C7	1.3608 (18)	C18—H18C	0.9800
C6—H6	0.9500	C19—H19A	0.9800
C7—C8	1.4144 (17)	C19—H19B	0.9800
C7—H7	0.9500	C19—H19C	0.9800
			
C2—O2—C18	117.22 (9)	O1—C11—C3	120.54 (11)
C8—O3—C19	117.70 (10)	C12—C11—C3	119.14 (10)
C2—C1—C10	120.89 (11)	C17—C12—C13	118.98 (11)
C2—C1—H1	119.6	C17—C12—C11	119.42 (10)
C10—C1—H1	119.6	C13—C12—C11	121.57 (11)
O2—C2—C1	124.50 (11)	C14—C13—C12	120.60 (12)
O2—C2—C3	115.09 (10)	C14—C13—H13	119.7
C1—C2—C3	120.37 (10)	C12—C13—H13	119.7
C4—C3—C2	118.84 (11)	C15—C14—C13	118.15 (12)
C4—C3—C11	118.84 (11)	C15—C14—H14	120.9
C2—C3—C11	122.32 (10)	C13—C14—H14	120.9
C3—C4—C5	122.10 (11)	F1—C15—C16	118.53 (12)
C3—C4—H4	118.9	F1—C15—C14	118.26 (12)
C5—C4—H4	118.9	C16—C15—C14	123.20 (12)
C4—C5—C6	122.91 (11)	C15—C16—C17	117.88 (12)
C4—C5—C10	118.57 (10)	C15—C16—H16	121.1
C6—C5—C10	118.52 (11)	C17—C16—H16	121.1
C7—C6—C5	120.93 (11)	C16—C17—C12	121.13 (11)
C7—C6—H6	119.5	C16—C17—H17	119.4
C5—C6—H6	119.5	C12—C17—H17	119.4
C6—C7—C8	120.28 (11)	O2—C18—H18A	109.5
C6—C7—H7	119.9	O2—C18—H18B	109.5
C8—C7—H7	119.9	H18A—C18—H18B	109.5
O3—C8—C9	124.86 (11)	O2—C18—H18C	109.5
O3—C8—C7	114.32 (10)	H18A—C18—H18C	109.5
C9—C8—C7	120.82 (11)	H18B—C18—H18C	109.5
C8—C9—C10	119.84 (11)	O3—C19—H19A	109.5
C8—C9—H9	120.1	O3—C19—H19B	109.5
C10—C9—H9	120.1	H19A—C19—H19B	109.5
C1—C10—C9	121.26 (11)	O3—C19—H19C	109.5
C1—C10—C5	119.10 (10)	H19A—C19—H19C	109.5
C9—C10—C5	119.61 (10)	H19B—C19—H19C	109.5
O1—C11—C12	120.27 (11)		
			
C18—O2—C2—C1	−8.47 (17)	C8—C9—C10—C5	−0.31 (17)
C18—O2—C2—C3	169.07 (10)	C4—C5—C10—C1	1.65 (16)
C10—C1—C2—O2	176.29 (10)	C6—C5—C10—C1	−178.51 (11)
C10—C1—C2—C3	−1.12 (18)	C4—C5—C10—C9	−179.93 (10)
O2—C2—C3—C4	−173.95 (10)	C6—C5—C10—C9	−0.08 (16)
C1—C2—C3—C4	3.70 (17)	C4—C3—C11—O1	43.90 (16)
O2—C2—C3—C11	5.21 (16)	C2—C3—C11—O1	−135.26 (12)
C1—C2—C3—C11	−177.14 (11)	C4—C3—C11—C12	−133.52 (12)
C2—C3—C4—C5	−3.63 (17)	C2—C3—C11—C12	47.32 (16)
C11—C3—C4—C5	177.18 (10)	O1—C11—C12—C17	28.70 (17)
C3—C4—C5—C6	−178.86 (11)	C3—C11—C12—C17	−153.88 (11)
C3—C4—C5—C10	0.98 (17)	O1—C11—C12—C13	−149.31 (12)
C4—C5—C6—C7	−179.94 (11)	C3—C11—C12—C13	28.11 (16)
C10—C5—C6—C7	0.22 (18)	C17—C12—C13—C14	0.55 (18)
C5—C6—C7—C8	0.02 (19)	C11—C12—C13—C14	178.57 (11)
C19—O3—C8—C9	5.92 (18)	C12—C13—C14—C15	1.42 (19)
C19—O3—C8—C7	−174.07 (11)	C13—C14—C15—F1	178.38 (11)
C6—C7—C8—O3	179.56 (11)	C13—C14—C15—C16	−2.2 (2)
C6—C7—C8—C9	−0.42 (18)	F1—C15—C16—C17	−179.69 (11)
O3—C8—C9—C10	−179.42 (10)	C14—C15—C16—C17	0.9 (2)
C7—C8—C9—C10	0.56 (17)	C15—C16—C17—C12	1.21 (19)
C2—C1—C10—C9	−179.95 (10)	C13—C12—C17—C16	−1.91 (18)
C2—C1—C10—C5	−1.55 (17)	C11—C12—C17—C16	−179.97 (11)
C8—C9—C10—C1	178.08 (11)		

### Hydrogen-bond geometry (Å, °)

**Table:**

Cg1 is the centroid of the C1–C5/C10 ring.

**Table:**

*D*—H···*A*	*D*—H	H···*A*	*D*···*A*	*D*—H···*A*
C18—H18B···Cg1^i^	0.98	2.85	3.7479 (14)	152
C17—H17···O1^ii^	0.95	2.55	3.2930 (16)	136
C18—H18A···O3^iii^	0.98	2.39	3.3603 (16)	169

Symmetry codes: (i) *x*, −*y*+1/2, *z*−1/2; (ii) −*x*, −*y*, −*z*; (iii) *x*−1, *y*, *z*−1.
